# *Lactiplantibacillus plantarum* WLPL04 from Human Breast Milk Attenuates Hyperuricemia via Coordinated Purine Salvage Pathway, Renal Transporter Regulation, and Gut Microbiota Remodeling

**DOI:** 10.3390/nu17213447

**Published:** 2025-10-31

**Authors:** Min Wei, Yingsheng Hu, Zhihong Zhang, Liang Qiu, Xueying Tao, Hua Wei

**Affiliations:** 1 State Key Laboratory of Food Science and Technology, Nanchang University, Nanchang 330047, China; 2 International Institute of Food Innovation Co., Ltd., Nanchang University, Nanchang 330047, China; 3 Centre for Translational Medicine, Jiangxi University of Traditional Chinese Medicine, Nanchang 330047, China

**Keywords:** hyperuricemia, *Lactiplantibacillus plantarum* WLPL04, xanthine oxidase, purine salvage pathway, gut microbiota

## Abstract

Background: Hyperuricemia (HUA), a metabolic disorder characterized by high serum uric acid (UA) level, presents a growing global health challenge. Method: In this study, a stable murine model of HUA was established by orally administering adenine (100 mg/kg) and potassium oxonate (600 mg/kg) in C57BL/6J mice, resulting in significant elevation of serum UA and xanthine oxidase (XOD) activity, as well as renal pathological alterations. Given the anti-hyperuricemia potential of *Lactiplantibacillus plantarum* WLPL04, a strain from a human breast milk was evaluated. Conclusions: Oral administration of *L. plantarum* WLPL04 significantly reduced serum UA level and XOD activity in a dose-dependent manner. Moreover, *L. plantarum* WLPL04 treatment enhanced UA excretion by upregulating *ABCG2* and downregulating *URAT1* and *GLUT9* expression. It ameliorated renal injury and suppressed inflammation via downregulation of the NLRP3 inflammasome pathway. 16S rRNA gene sequencing revealed that *L. plantarum* WLPL04 restored gut microbial diversity and promoted the enrichment of beneficial genera such as *Bacteroides,* which was negatively correlated with UA in serum, creatinine, and inflammatory cytokines. Moreover, transcript analysis revealed upregulation of purine salvage genes (*hpt* and *xpt*), suggesting enhanced salvage pathway recycling of purine bases and reduced urate production. Those findings suggest that *L. plantarum* WLPL04 exerted multi-targeted anti-hyperuricemia effects through coordinated regulation of host purine metabolism, urate transport, inflammation, and gut microbiota composition, providing a promising probiotic candidate for HUA management.

## 1. Introduction

Hyperuricemia (HUA), defined as an abnormally elevated serum uric acid (UA) level, is an increasingly prevalent metabolic disorder worldwide [1]. The incidence of HUA is rising worldwide, with high rates in developed regions, such as Japan (26.8%) [2] and the United States (21%) [3], while in China it has exceeded 13% [4] and continues to rise annually. As the direct precursor of gout, hyperuricemia is also associated with numerous chronic conditions, including kidney stones, cardiovascular diseases, type 2 diabetes, and metabolic syndrome, etc. [5]. Current pharmacological treatments for hyperuricemia, including xanthine oxidase (XOD) inhibitors such as allopurinol and febuxostat [6], are effective in lowering serum UA levels. However, their long-term use is often associated with adverse effects, including hepatotoxicity, hypersensitivity reactions, and cardiovascular risks [6]. Given these limitations, increasing attention has been directed toward non-pharmacological strategies that offer broader systemic benefits with fewer side effects.

UA is the final product of purine metabolism in human due to the absence of uricase [7], with approximately 70% derived from endogenous synthesis and 30% from dietary intake [8]. UA levels are primarily regulated by host liver, kidneys, and intestines, with nearly 30% of uric acid excreted through the intestinal tract [8]. Increasing evidence suggests that the intestine contributes to uric acid balance though direct excretion mediated by urate transporters and indirect regulation via gut microbiome of purine metabolism [9,10,11]. Specific gut bacteria, such as those encoding the purine-degrading enzyme uricase, can directly metabolize uric acid into the more soluble compound allantoin [12,13], promoting its excretion. Furthermore, the gut microbiome is a critical modulator of host inflammation, intestinal barrier integrity, and immune response [14,15].

Probiotic intervention has emerged as a promising non-pharmacological approach to restore intestinal homeostasis and manage metabolic disorders [16,17]. Partial strains of lactic acid bacteria (LAB), including *Lactiplantibacillus plantarum* LTJ1/LTJ48 and *L. plantarum* SQ001 [18,19], exerted anti-hyperuricemia effects in a previous study through multiple mechanisms as follows: dietary purines were metabolized through ribonucleoside hydrolase RihA-C in *L. plantarum* or the nucleoside hydrolase Nhy69 in *Lactobacillus aviarius* CML180, as well as related enzymes in other LAB, thereby reducing uric acid precursors [20,21,22]; LAB modulates intestinal urate transporters to promote uric acid excretion [23] and restore gut microbial diversity and enrich beneficial taxa, which contributes to uric acid degradation and metabolic homeostasis [23,24]. In addition, LAB-derived metabolites enhance intestinal barrier integrity and suppress inflammation [25], while some LAB strains reduce hepatic XOD or adenosine deaminase activity [26]. However, to our limited knowledge, systematic research elucidating the urate-lowering mechanisms of LAB is rare.

Therefore, in this study, the urate-lowering mechanisms of *L. plantarum* WLPL04 were elucidated by focusing on the transcriptional regulation of purine salvage pathway genes and the probiotic host interactions involving urate metabolism, inflammatory, and gut microbiota modulation.

## 2. Materials and Methods

### 2.1. Culture of L. plantarum WLPL04 Under Purine Substrate

*L. plantarum* WLPL04 from human breast milk was cultured in sterile deMan, Rogosa, and Sharpe agar (MRS broth; Beijing Solarbio Science & Technology Co., Ltd., Beijing, China) under anaerobic conditions at 37 °C for 12 h [27]. To assess the response of *L. plantarum* WLPL04 to purine substrates, the strain was cultured in MRS broth supplemented with either inosine or xanthine (Beyotime Institute of Biotechnology Co., Ltd., Jiangsu, China) at a final concentration of 1 mM and 2 mM, respectively, and incubated anaerobically at 37 °C for 24 h. Cells were collected by centrifugation at 6000× *g* for 10 min at 4 °C, and then washed twice with ice-cold phosphate-buffered saline (PBS). The collected pellets were immediately stored at −80 °C for later use in RNA extraction and gene expression analysis.

### 2.2. Hyperuricemia Modeling Design

Male Kunming (KM) mice and C57BL/6 (C57) mice were purchased from CRISBIO (Jiangxi, China) and acclimatized on a standard diet and fresh water under a 12 h light/dark cycle at a temperature of 23 ± 1 °C and relative humidity of 54 ± 2% for 1 week. The protocol was approved by Wuhan Myhalic Biotechnology Co., Ltd. Animal Experimentation Ethics Committee (HLK-20240617-002).

**Figure 1:**
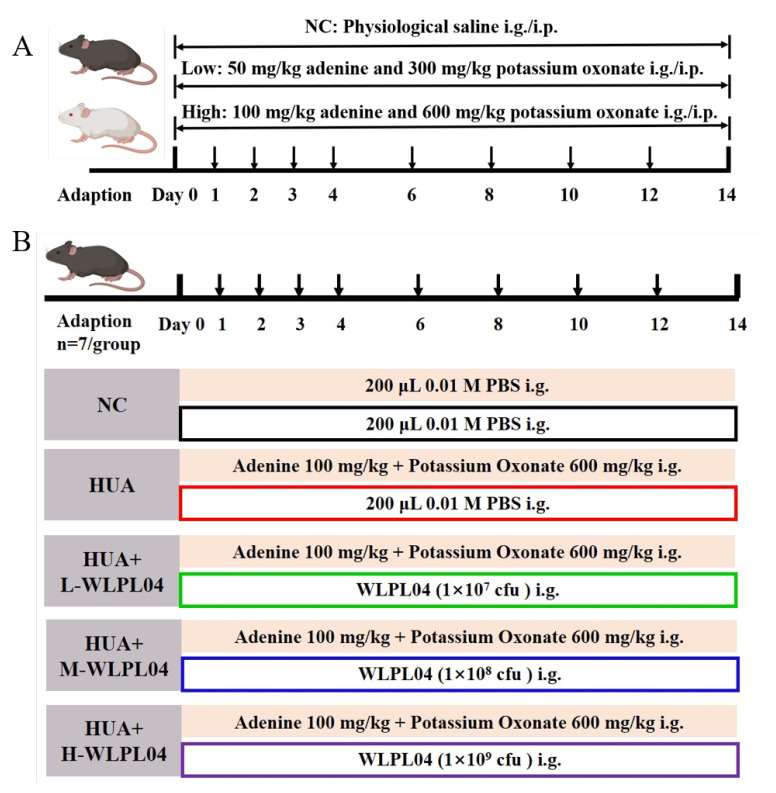
Experimental design for hyperuricemia (HUA) modeling. (**A**) Hyperuricemia induction protocol in Kunming (KM) and C57BL/6J (C57) mice via intragastric gavage (IG) or intraperitoneal injection (IP) of adenine and potassium oxonate (Low: 50 + 300 mg/kg; High: 100 + 600 mg/kg; NC: control). (**B**) WLPL04 intervention scheme in C57BL/6J HUA mice. NC: control; HUA: modeling agents; L-WLPL04/M-WLPL04/H-WLPL04: WLPL04 at 1 × 10^7^/1 × 10^8^/1 × 10^9^ CFU/day (n = 7/group).

To establish a stable hyperuricemia mouse model, different mouse strains (KM and C57, male, 18–20 g, n = 7 per group) were used [28]. Mice received either low-dose (50 mg/kg adenine and 300 mg/kg potassium oxonate) or high-dose (100 mg/kg adenine and 600 mg/kg potassium oxonate) formulations prepared in physiological saline. Two administration routes were used, oral gavage (i.g.) and intraperitoneal injection (i.p.). Mice were randomly assigned to 12 groups as illustrated in Figure 1A. Body weight was measured daily, and the gavage volume was adjusted according to the weight measured immediately before each administration.

### 2.3. L. plantarum WLPL04 Intervention in Hyperuricemia Model

As previously described, male C57BL/6J mice were acclimated for one week and then randomly assigned to five groups (n = 7/group), as shown in Figure 1B. Normal control group (NC) received 0.01 M PBS daily. The hyperuricemia model group (HUA) received adenine (100 mg/kg) and potassium oxonate (600 mg/kg) by oral gavage on days 0, 1, 2, 3, 4, 6, 8, 10, 12, and 14. The WLPL04-treated groups (HUA + L04, HUA + M04, and HUA + H04) received the same modeling protocol as the HUA group, along with WLPL04 at a dose of 1 × 10^7^, 1 × 10^8^, or 1 × 10^9^ CFU, respectively. In these groups, probiotic administration was performed on days 1 to 14 consistently 2 h after the modeling drug. Body weight and health status were monitored throughout.

### 2.4. Preparation of Specimens

On the 14th day, 2 h after the final drug administration, whole blood was collected from the orbital sinus under anesthesia prior to euthanasia. After clotting at room temperature for 60 min, the samples were centrifuged at 3000× *g* for 10 min at 4 °C. Then, the obtained serum was stored at −80 °C for subsequent analysis. The kidneys were rapidly excised on an ice plate and rinsed three times with ice-cold saline. Tissue from one whole kidney was immediately flash-frozen in liquid nitrogen and then stored at −80 °C for further analysis. In addition, fresh intestinal tract contents were collected and frozen in liquid nitrogen until assay.

### 2.5. Urine Collection

Urine samples were collected from each mouse on day 14 using individual metabolic cages (one mouse per cage) over a 12 h period without food but with free access to water. To avoid contamination, the bottom of each cage was rinsed with distilled water prior to use, and sterile tubes were used to collect urine. Samples were immediately centrifuged at 4 °C, 3000× *g* for 10 min to remove debris, and the supernatants were stored at −80 °C until biochemical analysis.

### 2.6. Serum Biochemical Assay

The frozen serum samples were thawed and brought to room temperature before measurement. Serum and urine levels of UA, blood urea nitrogen (BUN), and creatinine, as well as the activities of XOD and adenosine deaminase (ADA) in serum and livers were determined using commercial assay kits (Nanjing Jiancheng Bioengineering Institute, Nanjing, China) according to the manufacturers’ instructions. Serum levels of IL-1β, IL-6 and IL-10 were determined using the respective ELISA kits, and nitric oxide (NO) levels were quantified with NO assay kits (Beyotime, Shanghai, China) according to the manufacturer’s instructions.

### 2.7. Histopathological Analysis

At the end of the experiment, tissues from a whole kidney were collected, fixed with 4% paraformaldehyde (PFA) solution, followed by dehydration, embedding, and sectioning at a thickness of 4 μm. The tissue sections were then stained with hematoxylin and eosin (H&E); images were acquired using a Carl Zeiss LSM 510 UV laser scanning confocal microscope (Carl Zeiss, Oberkochen, Germany).

### 2.8. Real-Time Quantitative Polymerase Chain Reaction (RT-qPCR) Analysis

Total RNA was extracted from renal tissue using TRIzol reagent (Invitrogen, Carlsbad, CA, USA), and from *L. plantarum* WLPL04 using RNeasy Mini Kit (Qiagen, Hilden, Germany), according to the manufacturers’ protocols. cDNA was synthesized using PrimeScript^TM^ RT Reagent Kit with gDNA Eraser (Takara Biomedical Technology, Beijing, China) following the manufacturer’s instructions. Quantitative PCR (qPCR) was performed by an ABI 7900 HT fast real-time PCR system (Applied Biosystems, Foster City, CA, USA) using SYBR^®^ Premix Ex Taq II kit (Takara Biomedical Technology, Beijing, China). The primer sequences used for RT-qPCR are shown in Table 1. Gene expression levels were analyzed using the 2^−ΔΔCt^ method, with all transcript levels normalized to the baseline value of the NC group for mouse tissue samples and to the untreated WLPL04 control for bacterial samples.

### 2.9. 16 S Ribosomal RNA Gene Sequencing and Data Analysis

Fresh mouse fecal samples were immediately stored at −80 °C in refrigerators until DNA extraction. Total microbial DNA was extracted from frozen samples using QIAamp Fast DNA Stool Mini Kit (Qiagen, USA). DNA concentration and purity were verified prior to downstream processing. For the pre-experiment a specific region of the 16 S rRNA gene was amplified via PCR using a specialized primer set. The following V3–V4 regions of the 16 S rRNA gene amplicon PCR primers were used: 5′-ACTCCTACGGGAGGCAGCA-3′ (forward primer) and 5′-GGACTACHVGGGTWTCTAAT-3′ (reverse primer). The purified PCR products were quantified using a microplate reader (BioTek Flx800, Winooski, VT, USA). Sequencing libraries were prepared using TruSeq Nano DNA LT Library Prep Kit (Illumina, San Digeo, CA, USA) according to the manufacturer’s recommendations. Samples were sequenced using a paired-end strategy on an Illumina MiSeq/NovaSeq platform. The results data were analyzed with QIIME2 to evaluate alpha and beta diversities of samples based on the gglot2 package (Version 3.5.1.). The different bacterial features among groups were identified using metagenomeSeq and LEfSe analyses.

### 2.10. RAW264.7 Cells Co-Culture with WLPL04

RAW264.7 cells were obtained from the Institute of Cell Biology, Chinese Academy of Sciences (Shanghai, China). Cells were cultured in high-glucose Dulbecco’s Modified Eagle Medium (DMEM; Gibco, Grand Island, NY, USA) supplemented with 10% (*v*/*v*) fetal bovine serum (FBS, PAN-Biotech GmbH, Aidenbach, Germany) and maintained at 37 °C in a humidified incubator with 5% CO_2_. The effect of WLPL04 on the viability of RAW264.7 cells was determined using Cell Counting Kit-8 (CCK-8) (Beyotime Biotechnology, Shanghai, China). RAW264.7 cells (5 × 10^4^ cells/well) were cultured overnight in 96-well plates, which was followed by treatment with WLPL04 at different multiplicities of infection (MOI; 1:1, 1:10, and 1:100). After 24 h of incubation, cell viability was calculated according to the manufacturer’s instructions. The absorbance (Abs) at 450 nm was measured using a microplate reader (Varioskan Flash, Thermo Fisher Scientific, San Jose, CA, USA).

Cell viability (%) = Abs_sample_/Abs_control_ × 100%

In a separate experiment, RAW264.7 cells (5 × 10^5^ cells/well) were seeded in 6-well plates and incubated overnight, then treated with serial MOI of WLPL04 (1:1, 1:10, and 1:100) or LPS (1 µg/mL), respectively. After 24 h, NO levels were detected by Griess Reagent Assay Kit (Beyotime Institute of Biotechnology, Shanghai, China); IL-6 and IL-1β levels were determined using ELISA kits (Neobioscience, Shenzhen, China).

### 2.11. Statistical Analysis

All experimental data were shown as the mean ± standard deviation (SD). Multiple groups were tested by one-way analysis of variance (ANOVA) and analyzed by two-tailed unpaired Student’s t-test. A *p*-value < 0.05 was considered as statistically significant. All statistical analyses were carried out using GraphPad Prism v10.4.3 (Graph Pad Software, Inc, La Jolla, CA, USA).

## 3. Results

### 3.1. Effects of Different Models on Survival, Body Weight, and UA Metabolism on Mice

**Figure 2:**
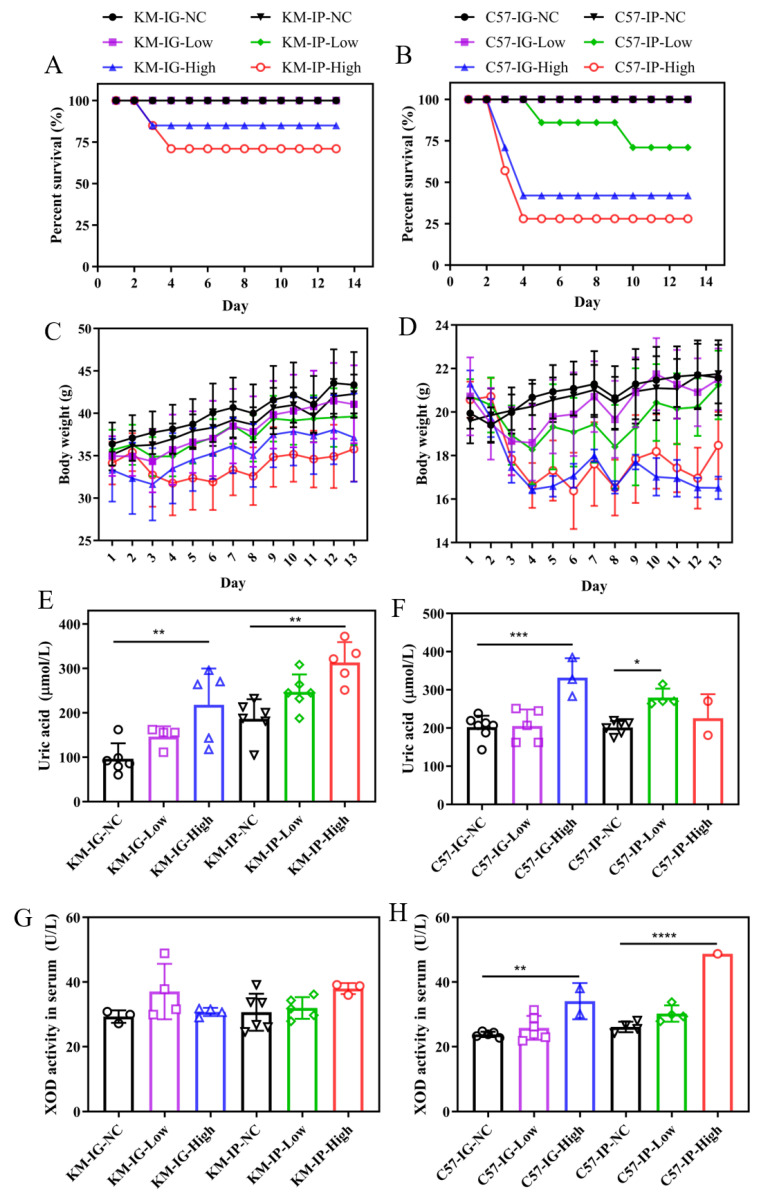
Survival, physiological parameters, and uric acid metabolism in HUA models. Survival rates of (**A**) KM and (**B**) C57 mice under different modeling conditions. Body weight changes in (**C**) KM and (**D**) C57 groups. Serum uric acid (UA) levels in (**E**) KM and (**F**) C57 mice. Xanthine oxidase (XOD) activity in (**G**) KM and (**H**) C57 mice. Statistical significance: * *p* < 0.05; ** *p* < 0.01; *** *p* < 0.001; **** *p* < 0.0001.

As shown in Figure 2A, the survival rates in KM mice dropped to 86% and 71% on day 4 in both high-dose groups and remained unchanged thereafter, while no deaths were observed in both low-dose groups. A similar trend was observed in C57 mice (Figure 2B), with survival rates dropping to 42% and 28% on day 4 in the two high-dose groups. However, deaths in the C57-IP-Low group began on day 5, and its survival ratio declined from 86% to 71%, while no deaths occurred in the C57-IG-Low group. KM mice in the IG groups showed relatively stable weight gain, while those in the IP groups, especially the IP-High group, experienced significant weight loss (Figure 2C). Similarly, C57 mice exhibited a comparable trend, with more dramatic weight loss in the IP-High group (Figure 2D). As shown in Figure 2E, high-dose groups of KM mice exhibited significantly elevated serum UA levels, reaching up to 218.12 ± 81.48 and 313 ± 45.72 μmol/L in the KM-IG-High and KM-IP-High groups. In C57 mice, serum UA levels were significantly higher in the C57-IG-High group (331.74 ± 51.01 μmol/L; *p* < 0.001) and C57-IP-Low group (279.81 ± 23.51 μmol/L; *p* < 0.05) compared with their respective controls (Figure 2F). XOD activity in KM mice (Figure 2G) exhibited relatively minor variation among all groups. Notably, both high-dose groups of C57 mice showed a significant increase in XOD activity (Figure 2H).

### 3.2. Renal Function Implications of Hyperuricemia Models

**Figure 3:**
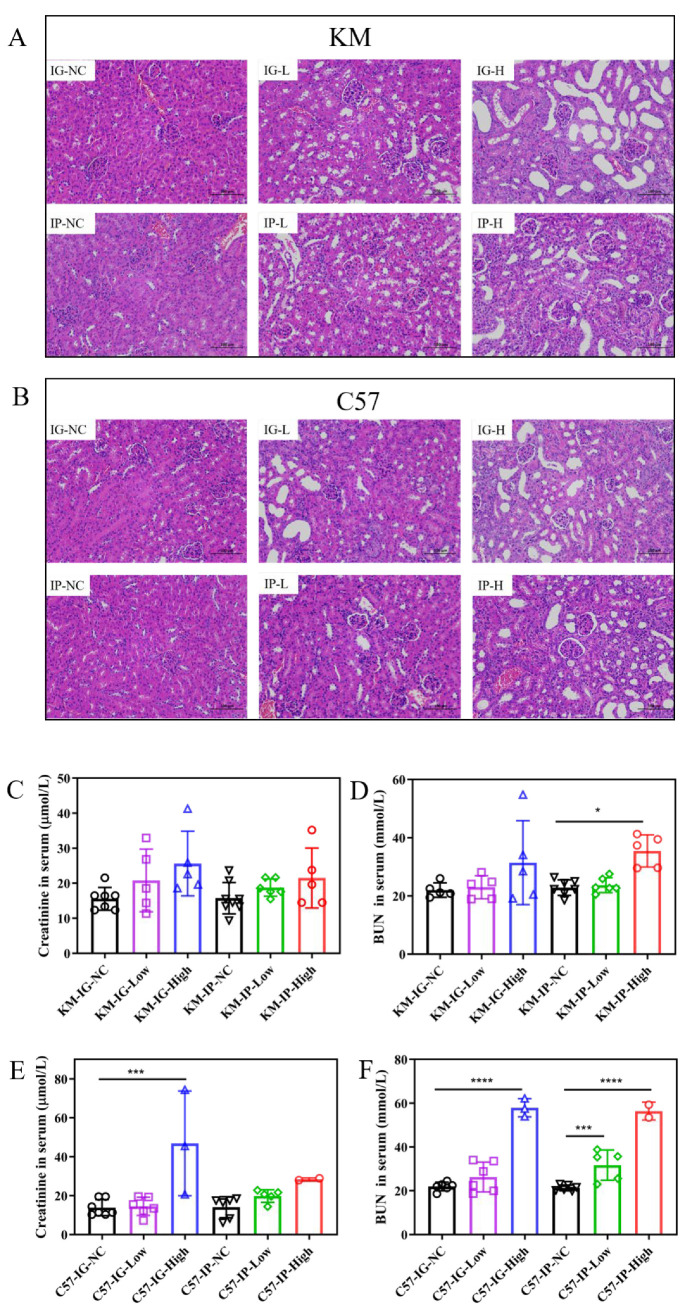
Renal impairment in HUA models. Representative H&E-stained kidney sections from (**A**) KM and (**B**) C57 mice (Scale bar = 100 μm). Serum creatinine and BUN levels in KM (**C**,**D**) and C57 mice (**E**,**F**). Statistical significance: * *p* < 0.05; *** *p* < 0.001; **** *p* < 0.0001.

Based on the established models, dose- and route-dependent renal damage was found in both KM (Figure 3A) and C57 (Figure 3B) mice. In KM mice, the IG-NC and IP-NC groups displayed normal renal histoarchitecture, whereas the IG-High and IP-High groups showed marked tubular dilation, epithelial shedding, and interstitial damage. Mild pathological changes were observed in the IG-Low and IP-Low groups. A similar trend was observed in C57 mice, with more severe lesions in the IG-High and IP-High groups compared with the IG-NC and IP-NC groups.

Serum creatinine and BUN are crucial clinical indicators of renal function. In Figure 3C,D, serum creatinine and BUN levels in KM mice were maintained at relatively stable levels compared with the NC group, suggesting a lesser degree of renal impairment. In contrast, significant elevations in both creatinine (Figure 3E) and BUN (Figure 3F) were observed in the C57-IG-High group, and BUN levels in C57-IP-Low and C57-IP-High were also significantly higher than those in the C57-IP-NC group (*p* < 0.0001).

These results suggested that the IG administration induced hyperuricemia with less physiological stress compared to the IP method in both models. Of all the groups, the C57-IG-High group was selected for subsequent intervention with LAB due to its significant UA elevation while maintaining relatively stable renal function and metabolic activity, providing a suitable model for investigating hyperuricemia mechanisms and assessing therapeutic interventions.

### 3.3. Expression of Purine Salvage Pathway Genes in L. plantarum WLPL04

**Figure 4:**
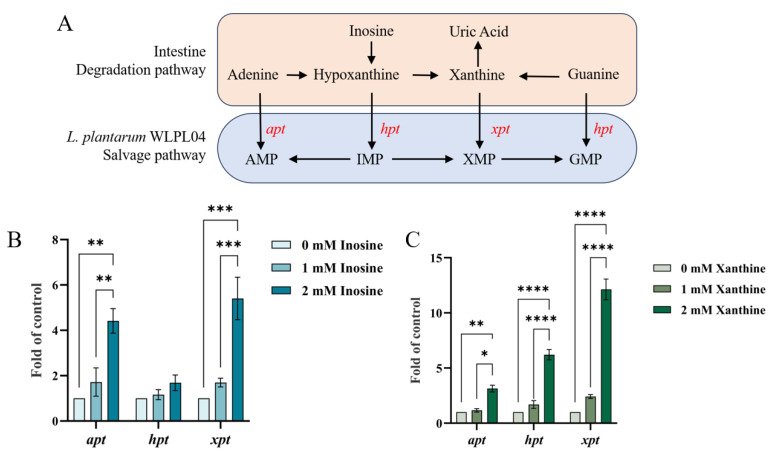
Purine salvage pathway activation in *L. plantarum* WLPL04. (**A**) Schematic of purine salvage genes in WLPL04. Relative mRNA expression of *apt*, *hpt*, and *xpt* under inosine (**B**) or xanthine (**C**) induction. Statistical significance: * *p* < 0.05; ** *p* < 0.01; *** *p* < 0.001; **** *p* < 0.0001.

*L. plantarum* WLPL04 possesses key purine salvage pathway genes, including hypoxanthine phosphoribosyltransferase (*hpt*), adenine phosphoribosyltransferase (*apt*), and xanthine phosphoribosyltransferase (*xpt*), which enable the reutilization of adenine, hypoxanthine, and guanine, thereby reducing the conversion of purines to UA in the intestine (Figure 4A). Functional validation revealed that 2 mM inosine upregulated the expression of *apt* and *xpt* by 4.4-fold and 5.4-fold, respectively (*p* < 0.01 and *p* < 0.001; Figure 4B), while *hpt* showed non-significant change (*p >* 0.05). In contrast, under 2 mM xanthine induction, the transcription levels of *apt*, *hpt*, and *xpt* were upregulated to 3.2-, 6.2-, and 12.1-fold higher than those in the control group, respectively (*p* < 0.0001; Figure 4C). Those results demonstrated that the purine salvage pathway was efficiently activated by *L. plantarum* WLPL04, thereby reducing the conversion of purines into urate. This metabolic feature highlighted the potential of *L. plantarum* WLPL04 to alleviate hyperuricemia, which was substantiated in subsequent in vivo experiments.

### 3.4. Effect of L. plantarum WLPL04 on Uric Acid and Physiological Parameters

**Figure 5:**
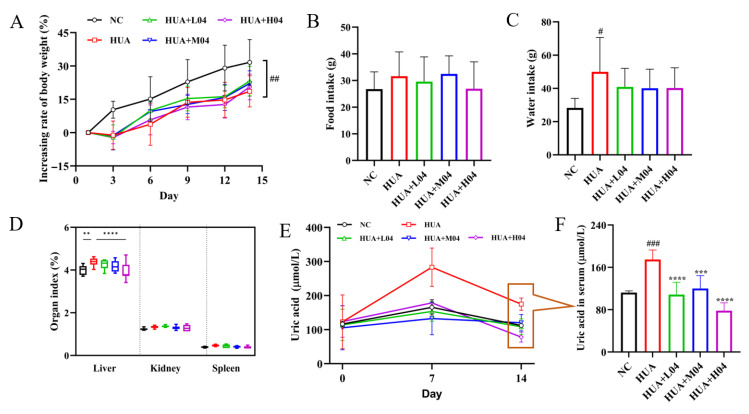
*L. plantarum* WLPL04 ameliorates systemic hyperuricemia. (**A**) Body weight changes. (**B**) Food intake. (**C**) Water consumption. (**D**) Organic index. (**E**) Time-course serum UA levels. (**F**) Serum UA on day 14. Statistical significance: ** *p* < 0.01; *** *p* < 0.001; **** *p* < 0.0001 vs. HUA group; # *p* < 0.1; ## *p* < 0.01; ### *p* < 0.001 vs. NC group.

As shown in Figure 5A, all groups showed a similar trend in body weight gain, but the HUA group exhibited a significantly lower body weight gain than the NC group (*p* < 0.01). Food intake was comparable among all groups (Figure 5B), while the excessive water consumption in the HUA group was partially alleviated by WLPL04 intervention (Figure 5C). Organ index analysis showed a significant increase in liver index in the HUA group (*p* < 0.01), which was significantly reversed in the intervention groups, particularly in the HUA + H04 group (*p* < 0.0001; Figure 5D). To assess the time-dependent effect of *L. plantarum* WLPL04 on uric acid metabolism, serum UA levels were measured on days 0, 7, and 14. As shown in Figure 5E, serum UA levels in the HUA group increased sharply by day 7, confirming successful induction of hyperuricemia. In contrast, all WLPL04-treated groups (L04, M04, and H04) exhibited a dose-dependent reduction in UA levels (Figure 5F), remaining significantly lower than those of the HUA group, as compared with those of the NC group.

### 3.5. Renal Protective Effect of L. plantarum WLPL04 in Hyperuricemia

**Figure 6:**
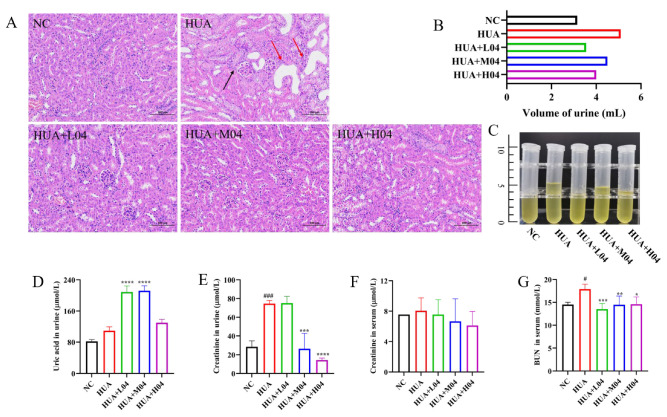
Renal protective effects of *L. plantarum* WLPL04. (**A**) H&E-stained kidney sections (Scale bar = 100 μm) (**B**) Urine volume. (**C**) 24 h urinary UA excretion. (**D**) UA levels in urine. (**E**) Urine creatinine. (**F**) Creatinine levels in serum (**G**) BUN levels in serum. Statistical significance: * *p* < 0.05; ** *p* < 0.01; *** *p* < 0.001; **** *p* < 0.0001 vs. HUA group; # *p* < 0.1; ### *p* < 0.001 vs. NC group.

To further assess the renal protective effect of *L. plantarum* WLPL04, renal histopathology and function markers were evaluated (Figure 6). As shown in Figure 6A, pronounced pathological changes were observed in the HUA group, including glomerular atrophy (black arrow) and tubular dilatation with inflammatory infiltration (red arrows). In contrast, those histological lesions were alleviated in all WLPL04-treated groups, with the HUA + H04 group exhibiting nearly normal renal architecture. Urine volume was significantly elevated in the HUA group compared with the NC group, reflecting renal dysfunction. This elevation was reversed by *L. plantarum* WLPL04 treatment in a dose-dependent manner (Figure 6B,C). Furthermore, urinary UA levels were significantly elevated in the HUA + L04 and HUA + M04 groups (*p* < 0.0001), indicating enhanced renal UA excretion (Figure 6D). However, urinary creatinine levels (Figure 6E) were markedly elevated in the HUA group (*p* < 0.001), suggesting renal impairment, but were significantly reduced by *L. plantarum* WLPL04 administration, particularly in the HUA + H04 group (*p* < 0.0001). Serum creatinine levels remained unchanged across all groups (Figure 6F), whereas BUN levels were significantly increased in the HUA group (*p* < 0.05) and restored to near-normal levels following *L. plantarum* WLPL04 treatment (Figure 6G).

### 3.6. Effects of L. plantarum WLPL04 on Inflammation

**Figure 7:**
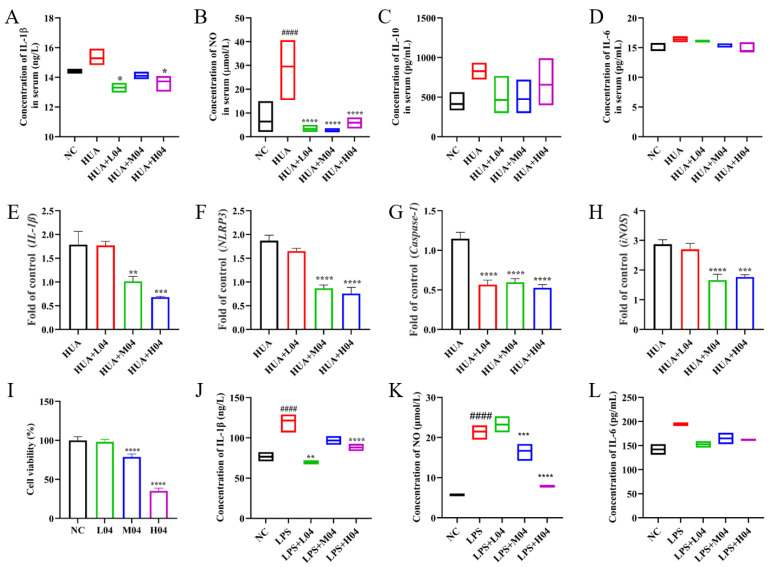
Anti-inflammatory mechanisms of *L. plantarum* WLPL04. Serum inflammatory markers (**A**) IL-1β, (**B**) NO, (**C**) IL-10, and (**D**) IL-6. Renal mRNA expression levels of (**E**) *IL-1β*, (**F**) *NLRP3*, (**G**) *Caspase-1*, and (**H**) *iNOS*. Viability of RAW264.7 cells co-cultured with WLPL04 (**I**), and in vitro the secretion of (**J**) IL-1β, (**K**) NO, and (**L**) IL-6 in LPS-stimulated macrophages. Statistical significance: * *p* < 0.05; ** *p* < 0.01; *** *p* < 0.001; **** *p* < 0.0001 vs. HUA group; #### *p* < 0.0001 vs. NC group.

To further elucidate the anti-inflammatory mechanisms of *L. plantarum* WLPL04 in hyperuricemia, both in vivo cytokine level and in vitro inflammatory responses were assessed (Figure 7). Serum levels of IL-1β (Figure 7A) and NO (Figure 7B) were significantly elevated in the HUA group compared with the NC group. *L. plantarum* WLPL04 treatment significantly reduced those inflammatory markers’ levels (*p* < 0.05; *p* < 0.0001, respectively). However, IL-10 (Figure 7C) and IL-6 (Figure 7D) levels showed no significant differences among all groups. Given the significant changes in serum IL-1β and NO levels, and in light of evidence that the NLRP3 inflammasome is a key mediator of urate-induced inflammation via caspase-1 activation and subsequent maturation of IL-1β [29], the expression levels of those related genes in renal tissue were assessed. As shown in Figure 7E–H, *L. plantarum* WLPL04 also markedly downregulated mRNA expression of *IL-1β*, *NLRP3*, *Caspase-1*, and *iNOS*.

To validate these effects, an in vitro model was established using RAW264.7 macrophages stimulated with LPS (1 μg/mL). As shown in Figure 7J,K, LPS significantly induced the production of IL-1β and NO, confirming activation of the inflammatory response. Co-culture with *L. plantarum* WLPL04 at different MOIs significantly reduced IL-1β (Figure 7J) and NO levels (Figure 7K), further supporting the anti-inflammatory effects of WLPL04 in macrophages.

### 3.7. Regulation of Uric Acid Metabolism by L. plantarum WLPL04

**Figure 8:**
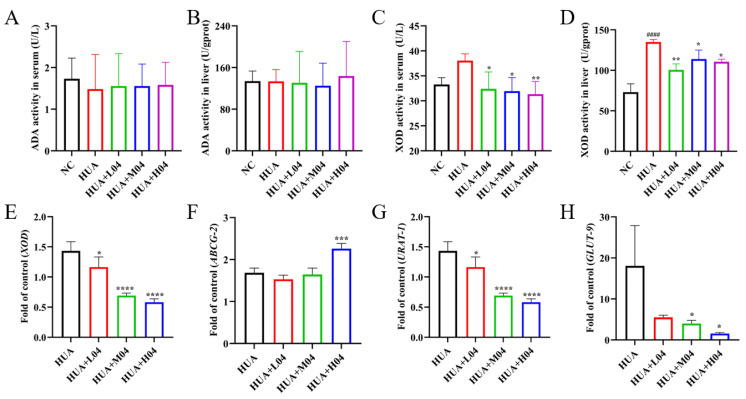
Regulation of uric acid metabolism by *L. plantarum* WLPL04. ADA activity in (**A**) serum and (**B**) liver. XOD activity in (**C**) serum and (**D**) liver. (**E**) Hepatic *XOD* mRNA expression. (**F**–**H**) Renal transporter mRNA: (**F**) *ABCG2*, (**G**) *URAT1*, (**H**) *GLUT9*. Statistical significance: * *p* < 0.05; ** *p* < 0.01; *** *p* < 0.001; **** *p* < 0.0001 vs HUA group; #### *p* < 0.0001 vs. NC group.

To investigate the effect of *L. plantarum* WLPL04 on UA metabolism, the activities of key purine-metabolizing enzymes and the expression of urate transporter genes were examined (Figure 8). As shown in Figure 8A,B, ADA activity in both the serum and liver showed no significant differences across groups, indicating that *L. plantarum* WLPL04 had little effect on purine catabolism via ADA pathway. XOD is an enzyme that catalyzes the conversion of hypoxanthine and xanthine to UA. Its activity was significantly elevated in both the serum and liver in the HUA group and markedly reduced by *L. plantarum* WLPL04 treatment (Figure 8C,D). Correspondingly, hepatic *XOD* mRNA expression also showed a dose-dependent reduction (Figure 8E), consistent with the observed inhibition of enzymatic activity.

Furthermore, the expression of urate transporters, which are essential for regulating UA reabsorption and excretion in the kidneys and intestine, was detected. ATP-binding cassette sub-family G member (ABCG-2) is a key transporter protein for UA excretion through the intestine and kidneys and was significantly upregulated by *L. plantarum* WLPL04 treatment (Figure 8F), suggesting enhanced urate excretion. In contrast, urate transporter 1 (*URAT1*; Figure 8G) and glucose transporter 9 (*GLUT9*; Figure 8H), two major resorptive transporters responsible in renal proximal tubules, were significantly downregulated by *L. plantarum* WLPL04 in a dose-dependent manner. Those findings indicate that *L. plantarum* WLPL04 not only inhibited UA synthesis through reducing XOD activity but also promoted its excretion by modulating the expression of multiple urate transporters.

### 3.8. L. plantarum WLPL04 Modulates Gut Microbiota

**Figure 9:**
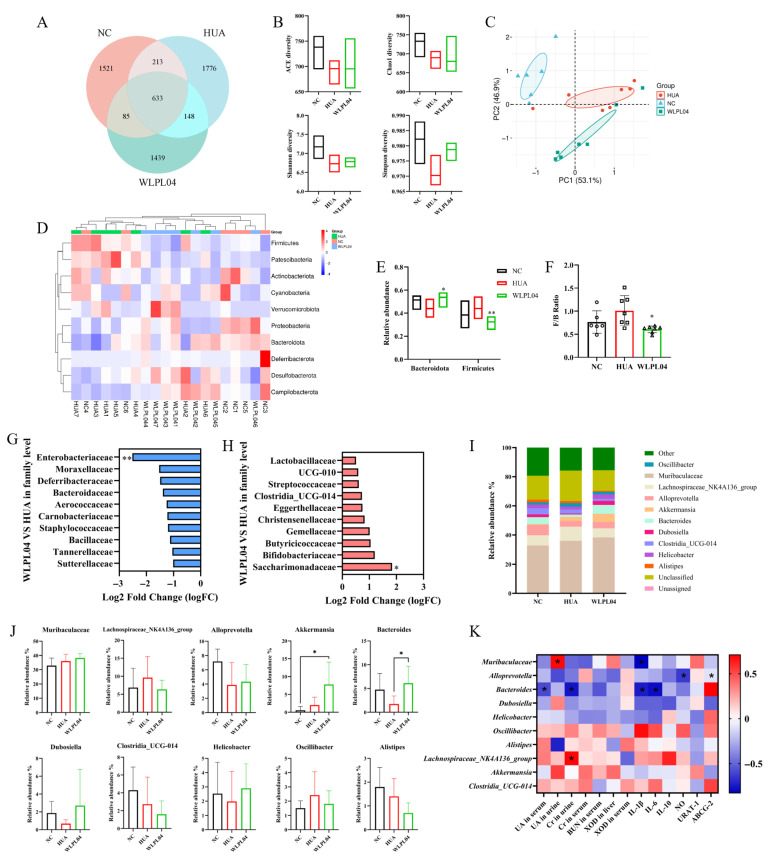
Gut microbiota remodeling by *L. plantarum* WLPL04. (**A**) Venn diagram of operational taxonomic units (OTUs). (**B**) Alpha diversity indices (ACE, Chao1, Shannon, Simpson). (**C**) PCoA plot based on Bray–Curtis distance. (**D**) Phylum-level composition. (**E**) The relative abundance of *Firmicutes* and *Bacteroidota* at the phylum level. (**F**) *Firmicutes*/*Bacteroidota* (F/B) ratio. (**G**) HUA-enriched families, (**H**) WLPL04-enriched families. (**I**) Stacked bar chart of genus-level abundances. (**J**) The relative abundance of genus level. (**K**) Correlation network between microbiota and HUA indices (red: positive; blue: negative). Statistical significance: * *p* < 0.05; ** *p* < 0.01.

The high-dose *L. plantarum* WLPL04 group was selected for gut microbiota analysis, as it exhibited the most pronounced therapeutic effects. As shown in Figure 9A, the WLPL04 group exhibited distinct operational taxonomic units (OTUs) compared with the NC and HUA groups. Alpha diversity indices further highlighted the dysbiosis in the HUA group with reduced ACE, Chao1, Shannon, and Simpson indices, whereas *L. plantarum* WLPL04 treatment restored these indices toward normal levels (Figure 9B). Principal coordinate analysis (PCoA) based on Bray–Curtis distances demonstrated distinct clustering of gut microbiota among groups. The HUA group displayed a pronounced shift away from the NC controls, whereas the WLPL04-treated group showed a partial shift toward NC (Figure 9C). At the phylum level, the HUA group exhibited an increase in *Firmicutes* and *Patescibacteria* (Figure 9D), whereas *L. plantarum* WLPL04 intervention elevated *Bacteroidota* and reduced *Firmicutes* (Figure 9E), and consequently lowered the *Firmicutes*/*Bacteroidota* (F/B) ratio (Figure 9F). Taxa enriched in the HUA group included *Enterobacteriaceae*, *Staphylococcaceae*, and *Deferribacteraceae* (Figure 9G). In contrast, *L. plantarum* WLPL04 treatment increased the abundance of beneficial families such as *Lactobacillaceae*, *Bifidobacteriaceae*, and *Christensenellaceae* (Figure 9H). At the genus level, the WLPL04 group exhibited higher abundance of *Bacteroides* and *Akkermansia*; conversely, *Oscillibacter* and *Clostridia_*UCG-014 were enriched in the HUA group (Figure 9I,J). Correlation analysis between intestinal microflora and hyperuricemia-related indices (Figure 9K) revealed that *Bacteroides* showed the strongest associations, correlating with UA, BUN, creatinine, and XOD levels. *Muribaculaceae*, *Akkermansia*, *Dubosiella*, and *Bacteroides* showed significant negative correlations with serum UA and pro-inflammatory cytokines (IL-1β, IL-6, and NO), whereas *Clostridia_*UCG-014 was positively correlated. These findings underscore the microbiota-mediated anti-hyperuricemia and anti-inflammatory potential of *L. plantarum* WLPL04.

## 4. Discussion

Hyperuricemia, a global metabolic disorder characterized by elevated levels of serum uric acid and associated renal and systemic inflammation, has attracted much attention related to exploring intervention therapies using probiotics, e.g., *L. paracasei* 259 [30], *Lacticaseibacillus paracasei* LT12 [31], *Lacticaseibacillus rhamnosus* Fmb14 [22], etc. In this study, multi-targeted anti-hyperuricemia effects were exerted by *L. plantarum* WLPL04 through coordinated regulation of purine metabolism, urate transport, inflammation, and gut microbiota composition, providing a promising probiotic candidate for HUA management.

Adenine combined with potassium oxonate—a widely used regimen—was employed to induce a stable hyperuricemia state. Adenine was metabolized by XOD to generate both uric acid and 2,8-dihydroxyadenine (DHA); the former contributes to systemic hyperuricemia, while the latter crystallizes in renal tubules, causing obstruction and inflammation [32]. Potassium oxonate further elevates SUA by inhibiting uricase-mediated degradation. Although this model reproduced the early metabolic and inflammatory features of human hyperuricemia, serum uric acid (SUA) levels remained below the clinical threshold (>7 mg/dL or ~416 µmol/L) due to residual uricase. Thus, the observed renal injury likely reflects combined effects of adenine-derived DHA crystals and mild hyperuricemia. Hyperuricemia models were constructed in KM and C57 mice in order to select one suitable model for verification of the anti-hyperuricemia effect of *L. plantarum* WLPL04. Although reductions in body weight and increased serum UA level and XOD activity were found in two models, as compared with KM mice, C57 mice exhibited stable profiles with better tolerance. To our limited knowledge, there have been no reports directly comparing these two mouse hyperuricemia models.

It was found that the expression of *apt* (3.2-fold, *p* < 0.01; 4.4-fold, *p* < 0.01), *hpt* (6.2-fold, *p* < 0.0001), and *xpt* (12.1-fold, *p* < 0.0001; 5.4-fold, *p* < 0.001) was significantly upregulated in mice treated with *L. plantarum* WLPL04 under xanthine and inosine conditions, respectively. Moreover, hepatic and serum XOD activity was significantly inhibited by *L. plantarum* WLPL04, thereby synergistically reducing UA production through both substrate diversion and enzymatic suppression. The enzymes HPT, APT, and XPT are involved in the purine salvage pathway, which reutilizes bases such as xanthine, hypoxanthine, adenine, and guanine for nucleotide synthesis [33,34]. In contrast, ADA and XOD are central enzymes of the purine degradation pathway, catalyzing the deamination of adenosine to inosine and the sequential oxidation of hypoxanthine and xanthine to UA, respectively. The capacity of several *Lactobacillus* species to absorb extracellular nucleosides and purine bases suggests their potential role in purine uptake, recycling, and host metabolic regulation [35,36]. Consistent with those findings, *Streptococcus thermophilus* DCC 2201 with the *hpt* gene was shown to lower serum UA levels in a hyperuricemia model (*p* < 0.05) [37], indicating a novel and sustainable strategy for hyperuricemia management by leveraging the innate purine-salvaging capabilities of *L. plantarum* WLPL04.

Urate excretion was significantly enhanced by upregulating *ABCG2* and downregulating *URAT1* and *GLUT9*, consistent with previously reported urate-lowering mechanisms of LAB; for instance, serum UA level was reduced by modulating *URAT1*, *GLUT9*, *PDZK1*, or *ABCG2* after *L. paracasei* LT12 and *L. paracasei* 259 interventions [30,31]. Therefore, *L. plantarum* WLPL04 demonstrated a comprehensive dual mechanism involving both inhibition of UA synthesis and enhancement of renal excretion.

XOD catalyzes the oxidation of hypoxanthine and xanthine to produce uric acid, concurrently generating reactive oxygen species (ROS) that directly activate the NLRP3 inflammasome [38]. Excessive accumulation of uric acid in serum further leads to crystal formation, which provides an additional stimulus for NLRP3 activation [39]. Together, XOD-derived ROS and urate crystals synergistically drive inflammatory progression in hyperuricemia. In this study, serum IL-1β and NO levels were significantly lowered by *L. plantarum* WLPL04 in HUA mice, accompanied by downregulation of mRNA expression of renal *NLRP3*, *Caspase-1*, and *IL-1β*, indicating suppression of systemic inflammation. This effect was further confirmed through an in vitro assay, namely, *L. plantarum* WLPL04 significantly suppressed the production of pro-inflammatory mediators (NO and IL-1β) in LPS-stimulated RAW264.7 macrophages. A similar anti-inflammatory effect was reported for *L. rhamnosus* Fmb14, which decreases serum TNF-α and IL-1β levels in HUA mice [22], suggesting that modulation of inflammasome pathways may be a common mechanism through which LAB exert anti-inflammatory effects in HUA.

Accumulating evidence has indicated the important role of gut microbiota in purine metabolism and systemic inflammation, both of which are central to HUA pathophysiology [15]. *L. plantarum* WLPL04 restored both microbial richness and evenness and decreased the F/B ratio, which is a widely accepted indicator of metabolic gut dysbiosis [40,41]. Microbiota profiling revealed that *L. plantarum* WLPL04 enriched beneficial families, e.g., *Lactobacillaceae*, *Bifidobacteriaceae*, and *Christensenellaceae*, known for their anti-inflammatory and short-chain fatty acid (SCFAs)-producing capacities [42,43], while reducing HUA-associated pro-inflammatory taxa such as *Enterobacteriaceae* and *Staphylococcaceae*, which are frequently linked to endotoxemia and gut barrier disruption [44,45]. In contrast to these studies, *Muribaculaceae*, *Akkermansia*, *Dubosiella*, and *Bacteroides* have been shown to be significant negative correlations with serum UA and pro-inflammatory cytokines (IL-1β, IL-6, and NO), whereas *Clostridia_UCG-014* and *Oscillibacter* were positively correlated with pro-inflammatory states by *L. plantarum* WLPL04 intervention. Previous reports showed that *Bacteroides* and *Akkermansia* enhanced gut barrier integrity and promoted purine degradation [46,47,48] and *Muribaculaceae* contributed to dietary purine degradation and SCFA production [49,50], thereby ameliorating HUA. This coordinated modulation not only restored gut microbial homeostasis but also directly linked microbiota shifts with improvements in UA metabolism, renal function, and systemic inflammation. To our limited knowledge, this is the first report to demonstrate that probiotic-driven enrichment of *Bacteroides* and *Akkermansia* acts in concert with the purine salvage pathway to alleviate hyperuricemia, providing a novel microbiota-based mechanism for HUA management. It would be worthwhile in future studies to explore uricase-knockout or urate-crystal models, and possibly human trials, to evaluate whether WLPL04 maintains its efficacy under more physiologically relevant hyperuricemia conditions.

## 5. Conclusions

*L. plantarum* WLPL04 from human breast milk effectively ameliorated hyperuricemia through a coordinated multi-target mechanism. It reduced UA production by significantly upregulating purine salvage pathway genes (*xpt* and *hpt*) and inhibiting serum and liver XOD activity, while enhancing UA excretion by upregulating *ABCG2* and downregulating *URAT1* and *GLUT9*. Moreover, it downregulated the expression of the inflammatory factors IL-1β and NO by inhibiting the NLRP3 inflammasome and restored gut microbiota diversity and enriched beneficial genera, including *Bacteroidetes*, whose abundance was closely related to decreased serum urate levels and inflammatory markers. Overall, those findings highlight *L. plantarum* WLPL04 as a promising probiotic candidate for the dietary management of hyperuricemia.

## Figures and Tables

**Table 1:** Primers used for qPCR.

Genes	Forward Primer (5′-3′)	Reverse Primer (5′-3′)
*apt*	TGGTTTTGCACCTGCTCGTA	TAGCACATTTTGTCCCGGCT
*xpt*	GGCGGTTCAAGGGATGTTTG	CTAAACGGACACCCCGATCA
*hpt*	CGTACTCAAGGGTGCCGTTT	ACATCACGACCCGTCACATC
*16s*	AGAGTTTGATCCTGGCTCAG	CTACGGCTACCTTGTTACGA
*iNOS*	GCGAAAGGTCATGGCTTCAC	TCTTCCAAGGTGCTTGCCTT
*IL-1* *β*	GCAACTGTTCCTGAACTCAACT	ATCTTTTGGGGTCCGTCAACT
*NLRP-3*	CCTGGGGGACTTTGGAATCAG	GATCCTGACAACACGCGGA
*Caspase-1*	ATCTTTCTCCGAGGGTTGG	AAGTCTTGTGCTCTGGGCAG
*XOD*	ATGACGAGGACAACGGTAGAT	TCATACTTGGAGATCATCACGGT
*ABCG2*	AGAATAGCATTAAGGCCAGGTTT	AGAATAGCATTAAGGCCAGGTTT
*GLUT9*	TGGACTCAATGCGATCTGG	AGAGAAGATAGCAGCCAGTGTTT
*URAT1*	CCGCTTCCGACAACCTCA	CTTCTGCGCCCAAACCTATC
*β-actin*	GCTCCTCCTGAGCGCAAGTA	CAGCTCAGTAACAGTCCGCC

## Data Availability

The original contributions presented in this study are included in the article. Further inquiries can be directed to the corresponding author.
